# Skills, attitude and knowledge for early-career hematologists: a pragmatic, scientific framework

**DOI:** 10.46989/001c.150336

**Published:** 2025-12-02

**Authors:** Mohamad Mohty, Yishan Ye, Rahul Banerjee

**Affiliations:** 1 Sorbonne Université; Centre de Recherche Saint-Antoine, INSERM UMRs 938; Service d’Hématologie Clinique et de Thérapie Cellulaire, Hôpital Saint Antoine, AP-HP, Paris, France.; 2 Bone Marrow Transplantation Center, The First Affiliated Hospital, Zhejiang University School of Medicine, 310000 Hangzhou, China; 3 Fred Hutchinson Cancer Center, Seattle, WA, USA

**Keywords:** Skills, Early-career, attitude, Knowledge

Hematology today is defined by accelerating discovery, from molecular stratification and targeted agents to cellular therapies and real-world evidence. With this expansion comes a corresponding obligation for academic hematologists: impact must be demonstrated rather than inferred. For trainees and junior faculty, good intentions and effort are insufficient unless they translate into safer care, more reliable processes, and more informative science. A triangular framework – with skills, attitude and knowledge as the vertices – offers a compact way to operationalize that translation successfully. *Skills* denote the day-to-day behaviors that make results dependable. *Attitude* describes the professional stance that preserves momentum, integrity, and resilience. *Knowledge* encompasses both what is learned and how it is converted into better decisions.

**Skills: turning intent into reproducible output**. Progress begins when objectives are expressed in observable terms. A unit that aims to “improve day-hospital chemotherapy flow” will not progress as quickly as one that states an expected reduction in delays beyond a defined threshold, an expected increase in resolved same-day toxicity calls without emergency referral, and a defined target for trial screening and enrollment. When goals are explicit and auditable, ambiguity recedes and early course correction becomes possible.

Time control is the second operational competence. In busy services, attention is the scarcest resource, and without deliberate protection it is consumed by interruptions. This is a challenge for academic hematologists and oncologists, but best practices for academic medical centers to optimize research productivity among their faculty have been established.^1^ Regardless, a few skills can go a long way in setting academic hematologists up for success. Clinical hours should be guarded from nonessential meetings, and analytic work (eg. writing, protocol design, data review etc.) requires uninterrupted blocks. Simple practices, such as batching administrative communication and insisting on meetings that end with a decision, shorten feedback loops and reduce errors introduced by context switching. On the other hand, when working with mentors, setting up regular scheduled meetings with a varying agenda of action items for discussion at each meeting (e.g., grant proposals or ongoing manuscripts) may be more successful than relying on asynchronous emails alone. The result of these steps is not only more output but *safer* output: hurried environments produce preventable toxicity misclassification, incomplete documentation, rejected manuscripts, and more.

Reasoning discipline matters as much as domain knowledge. High-stakes choices (eg. transplant candidacy in borderline physiologies, dose intensity in frail patients, the timing and sequencing of immune-based therapies, etc.) are vulnerable to overconfidence and hindsight bias. Attaching explicit probabilities to diagnostic and therapeutic judgments and reviewing calibration over time curbs these errors. Brief “premortems” – where a group imagines a hypothetical poor outcome and brainstorms strategies to have prevented it – make failure modes discussable before they occur.^2^ Statistical prompts help people avoid giving too much importance to rare but striking situations. Even “quality projects” benefit from design hygiene: prespecifying outcomes, favoring controlled before-and-after or interrupted time-series designs over naive pre/post comparisons, and documenting deviations. Such habits prevent spurious conclusions and increase the credibility of change proposals.

Communication is the final operational multiplier. Handovers that identify a single next action, a single owner, and a deadline reduce drift and clarify accountability. Decision-first writing stating the required decision up front and supporting it with succinct data, shortens time to action. Data displays that show absolute effects with denominators and a plain-language bottom line reduce misinterpretation. In manuscripts and protocols, consistent structure is not cosmetic; it is a safety feature that helps readers verify methods and reproduce results. Standardized communication reduces decision latency and error. Even when a hematologist’s identity is masked (i.e., through peer review), a structured approach and respectful writing style are essential to ensure that one’s suggestions are adopted by the authors to improve the manuscript’s readability and relevance to the hematology field at large.^3^

**Attitude: sustaining performance under pressure**. Technical competence does not persist without a professional stance that supports it. Integrity and accountability are foundational. Pre-specifying analyses when feasible, documenting changes to plans, declaring conflicts of interest, and protecting patient privacy are not administrative burdens: they are conditions of trust that allow results to be acted upon. Credibility accumulates slowly and can be lost suddenly. Small lapses have outsized downstream consequences.

Resilience is better conceived as a system than as a trait. Visible objectives, regular reviews with a mentor or peer, and transparent reporting create external structure that sustains motivation when workload is heavy, or results are delayed. Rejection of grants, manuscripts, or protocols should be normalized as variance inherent to ambitious work rather than personalized as failure. The appropriate response is disciplined iteration guided by specific reviewer signals rather than defensive rationalization.

Confidence should be calibrated rather than asserted. Ambition paired with explicit error budgets and contingency plans allows for principled risk-taking without compromising safety. Innovation in care pathways, trial screening rules, or toxicity monitoring is most effective when accompanied by predefined monitoring, escalation criteria, and early stopping rules. In this way, the downside is limited while the upside, a more efficient process or clearer evidence, remains substantial.

Local norms and system design strongly determine clinician behavior more than willpower. Teams that value measurement, feedback, and reproducibility make high standards the path of least resistance. Institutional rules should be understood. Within those boundaries, improvement efforts should be judged by whether they raise quality and equity. Not all colleagues will welcome change, particularly when it exposes process deficits. Progress depends on maintaining collegiality while keeping attention on patient-level outcomes and methodological clarity.

**Knowledge: building judgment that compounds**. Knowledge acquisition benefits from structure. A balanced curriculum for early-career hematologists includes core science, clinical evidence and toxicity science, methods for study design and causal inference, and systems science encompassing quality, safety, informatics, and health economics. Regular review works well in fields where learning relies on memorization. For the rest, reviewing new material soon after learning helps strengthen understanding and reveal what is still unclear. The ultimate test of knowledge is whether it changes decisions. A major paper is best converted into a short practice brief that specifies what, if anything, should change in the clinic the next day. A registry analysis is most valuable when it yields a concrete screening rule that identifies real patients for real studies. A toxicity pathway prevents harm when rendered as a checklist at defined time points rather than as a conceptual diagram. For junior faculty, advancing research sequentially over one’s career from clinical anecdotes to retrospective observations to prospective analyses is fundamental to establish one’s niche as a hematologist and also to advance information for the field. Even components of hematology that we now take for granted, for example the Rai staging system for chronic lymphocytic leukemia, once stemmed from such humble roots.^4^ On the other hand, knowledge that is not designed as a step toward modifying hematologists’ behavior will forever remain information rather than improvement.

**Measurement and feedback: closing the loop**. Routine review of a small set of indicators stabilizes standards and makes progress visible. At the patient level, avoidable emergency visits, the timeliness of treatments, and the capture of serious toxicities reveal whether safety is improving. At the process level, screened-to-enrolled conversion, protocol deviations, and data completeness indicate whether research operations are reliable. Team measures such as turnaround time on decisions and adherence to handover standards reflect coordination quality. Individual measures (eg. protected time honored, commitments delivered, skills acquired) track personal execution. Publishing these results within the team invites help where needed and reduces reliance on impressionistic assessments.

In summary, for younger hematologists, sustained impact depends less on exceptional talent than on consistent, measurable behaviors supported by professional stance and informed by structured learning. The combination of skills, attitude, and knowledge provides a concise three-point scientific framework for success. When goals are explicit, time and attention are protected, reasoning and communication are disciplined, integrity is visible, risk is managed, and knowledge is translated into action, variability falls and outcomes improve. The approach is sober by design: it favors evidence over rhetoric and systems over slogans. Applied faithfully, it aligns personal development with the obligations of modern hematology: safer care, stronger science, and more reliable teams.

## STATEMENTS AND DECLARATIONS

The authors declare no competing financial interests in relation with this work.

## ETHICAL APPROVAL

Not applicable.

## CONSENT TO PARTICIPATE/INFORMED CONSENT

Not applicable.

## CONSENT FOR PUBLICATION

Not applicable.
